# Factors Associated With Employment and Quality of Working Life in Patients With Metastatic Breast Cancer

**DOI:** 10.1002/cam4.71074

**Published:** 2025-07-27

**Authors:** Alina Kias, Martina E. Schmidt, Anouk E. Hiensch, Dorothea Clauss, Evelyn M. Monninkhof, Mireia Pelaez, Jon Belloso, Nadira Gunasekara, Maike G. Sweegers, Mark Trevaskis, Helene Rundqvist, Jana Müller, Joachim Wiskemann, Elsken van der Wall, Neil K. Aaronson, Milena Lachowicz, Ander Urruticoechea, Eva M. Zopf, Wilhelm Bloch, Martijn M. Stuiver, Yvonne Wengström, Anne M. May, Karen Steindorf

**Affiliations:** ^1^ German Cancer Research Center (DKFZ) and National Center for Tumor Diseases (NCT) Heidelberg (a Partnership Between DKFZ and University Medical Center Heidelberg) Heidelberg Germany; ^2^ Medical Faculty University of Heidelberg Heidelberg Germany; ^3^ Julius Center for Health Sciences and Primary Care University Medical Center Utrecht, Utrecht University Utrecht the Netherlands; ^4^ German Sport University Cologne Cologne Germany; ^5^ Gipuzkoa Cancer Unit, OSID‐Onkologikoa, BioGipuzkoa, Osakidetza San Sebastian Spain; ^6^ Faculty of Health Sciences Universidad Europea del Atlántico Santander Spain; ^7^ Netherlands Cancer Institute Amsterdam the Netherlands; ^8^ Mary MacKillop Institute for Health Research Australian Catholic University Melbourne Australia; ^9^ Karolinska Institutet Stockholm Sweden; ^10^ Working Group Exercise Oncology, Division of Medical Oncology National Center for Tumor Diseases (NCT) and Heidelberg University Hospital Heidelberg Germany; ^11^ Medical University of Gdańsk Gdańsk Poland; ^12^ Cabrini Cancer Institute Cabrini Health Melbourne Australia

**Keywords:** employment, exercise, fatigue, metastatic breast cancer, pain, quality of working life

## Abstract

**Purpose:**

As survival of patients with metastatic breast cancer (MBC) improves, their work situation is gaining importance. The aim of the current study was to identify factors associated with work status and quality of working life (QWL) in patients with MBC. Additionally, we investigated the effects of an exercise intervention on work status.

**Methods:**

Within the multinational PREFERABLE‐EFFECT exercise trial, 287 patients with MBC of working age (18–65 years) reported on their working situation over 9 months as a secondary endpoint. Among a subgroup of participants, QWL was assessed by the Quality of Working Life Questionnaire for Cancer Survivors (QWLQ‐CS) (*N* = 59).

**Results:**

At baseline, 157 (54.7%) participants were employed, of whom one‐third reported having recently reduced their amount of work because of fatigue (41.7%), cognitive problems (33.3%), or inability to meet work demands (33.3%). Participants wished for more flexible working hours (29.2%) and less productivity pressure (37.5%). Participants were less likely to work if they experienced higher levels of pain (*p* = 0.014). Among working participants, an academic education and higher levels of psychological distress were associated with a higher number of working hours (all *p* < 0.05). Fatigue, an academic education, and performing mentally strenuous tasks at work were negatively associated with QWL (all *p* < 0.05). The exercise intervention did not affect the number of hours worked during the study.

**Conclusions:**

Symptom management might be important for patients' ability to work. To help patients stay employed and improve QWL, employers should consider offering more flexible work arrangements and adapting to their employees' changing needs and abilities.

**Trial Registration:** The PREFERABLE‐EFFECT trial was registered with ClinicalTrials.gov on October 9, 2019 (NCT04120298).

## 1 Introduction

While metastatic breast cancer (MBC) remains an incurable disease, survival rates have improved over the last decades [1, 2]. As a result, the topic of working life is gaining importance for patients with MBC. In general, many patients reduce their working hours or put their jobs on hold after being diagnosed with cancer [3, 4, 5]. Those who continue working have reported being limited in the kinds of work they can do and having to reduce their working hours [6]. Past research has shown that the reasons for this are manifold. For instance, patients have reported symptoms such as sleep issues, cognitive problems, or fatigue [3, 4, 7, 8, 9]. Younger age, being single, and a higher education can increase the likelihood of employment [3, 4, 9, 10] whereas certain disease‐related variables are associated with a lower likelihood of employment, including chemotherapy [3, 9] or advanced tumor stage [9, 11] Furthermore, work‐related variables like type of job may play a role [3, 9, 12]. Residency, too, might influence employment due to differences in government policies and health insurance systems [13]. The majority of studies on factors associated with work status have been performed in the curative setting, whereas only a few studies have focused on patients with advanced stages of cancer.

For those patients who remain employed, it is important to understand which factors influence the quality of their working life (QWL). QWL represents the experiences and perceptions of a person in the work environment and has been found to be reduced in cancer survivors compared to healthy individuals [14]. Previous research has found better QWL in patients with cancer to be associated with higher perceived cognitive function [15], lower levels of depression [15, 16] and less fatigue [16]. Having received chemotherapy has been associated with lower QWL [17]. Work‐related variables also play an important role. For example, social support in the workplace by supervisors or colleagues is associated with a higher QWL [16, 18]. Furthermore, while QWL improves with managerial positions and higher income, physically demanding work seems to be detrimental [17]. However, research on QWL in patients with MBC is still scarce. To the best of our knowledge, a cross‐sectional study by Chapman et al. [15] is the only study that has directly investigated QWL in patients with MBC. In their study, better cognitive functioning and fewer depressive symptoms were associated with better QWL. Likewise, symptom burden has been identified as a major factor impacting work outcomes such as productivity for patients with MBC [19]. Elaborating further on these findings is important for providing a sound knowledge base for the development of effective measures for improving employment and QWL for patients with MBC. In the present research, we therefore investigated the association of work status and QWL with socio‐demographic, psychological, disease‐ and work‐related variables in patients with MBC. Additionally, self‐reported reasons for stopping working and patients' wishes for their work situation were analyzed.

Moreover, interventions that aimed at improving employment or work ability in patients with cancer have yielded mixed results so far [20, 21, 22]. Past studies investigated physical, psychosocial, and, to a small extent, work‐focused interventions and indicated only multidisciplinary interventions might be effective in promoting return to work [20, 22]. However, a recent meta‐analysis suggests that physical activity can also increase return to work after a cancer diagnosis [23]. One potential pathway may be the improvement of cancer‐related side effects like fatigue, which is associated with lower employment [4, 7] and can be targeted with exercise [24]. The PREFERABLE‐EFFECT study recently showed that supervised exercise could improve fatigue and quality of life (primary outcomes) in patients with MBC across several European countries and Australia, while it was also associated with reduced productivity losses, e.g., less short‐term sick leave [25]. Therefore, we also examined the effect of the PREFERABLE‐EFFECT exercise intervention on working volume (secondary outcome) in patients with MBC.

## 2 Materials and Methods

### 2.1 Design and Participants

The PREFERABLE‐EFFECT study was a randomized controlled exercise trial examining the effects of a 9‐month exercise intervention in patients with MBC across eight centres in Germany, the Netherlands, Spain, Sweden, Poland, and Australia. The trial was designed to examine health‐related quality of life and physical fatigue as primary endpoints and work, including QWL, as one of several secondary endpoints. The trial was funded by the European Union and the Australian Government and has been registered at ClinicalTrials.gov under the ID: NCT04120298. To be eligible for the study, patients had to be at least 18 years of age, be diagnosed with stage IV breast cancer, and have an ECOG (Eastern Cooperative Oncology Group scale) performance status ≤ 2. Additionally, they had to have a life expectancy > 6 months and no unstable bone metastases. A complete list of inclusion and exclusion criteria was published elsewhere [26]. In the present analyses, we limited the sample to participants of working age (18–65 years).

### 2.2 Intervention

A detailed description of the intervention has been published elsewhere [26]. In brief, participants randomized to the intervention group participated in a 9‐month exercise program that consisted of balance, resistance, and moderate‐to‐high intensity aerobic exercises. For the first 6 months, the intervention included two supervised exercise sessions per week. Each session lasted for 60 min. For the last 3 months, one supervised session was replaced by an unsupervised exercise session. The control group received care as usual and written information on the current physical activity guidelines for patients with cancer. All patients received an activity tracker (Fitbit) at baseline.

### 2.3 Outcomes

At baseline, 3‐, 6‐, and 9‐month, participants were asked about their employment status (“Do you have a paying job?”), current number of working hours per week and if they had reduced work in the previous 3 months. If they had reduced their working hours, they were asked to provide reasons and what they would have wished for with regard to their work arrangement by choosing up to three options each from a predefined list. There was a free text option for reporting other factors not listed. Additionally, participants rated the extent to which they performed mentally strenuous tasks, physically strenuous tasks, assigned tasks (i.e., carrying out a delegated task) and management tasks (i.e., planning, organization, decision‐making and control) in their job on a scale ranging from *1 (never)* to *5 (always)*. The questionnaire is available in S1. As an add‐on in four study centres (Germany, Australia, the Netherlands and Spain), participants were asked if they had worked in the last 4 weeks and, if so, to complete the Quality of Working Life Questionnaire for Cancer Survivors and its subscales, with scores ranging from 0 to 100 (higher scores representing better QWL) [14].

### 2.4 Potential Factors Associated With Work and QWL

Patient‐reported outcomes were assessed using validated questionnaires. Variables of interest included cancer‐related fatigue (EORTC QLQ‐FA12, range 0–100, higher scores indicating higher fatigue) [27], as it has been previously associated with employment and QWL [4, 16, 28], pain (EORTC QLQ‐C30 subscale, range 0–100, higher scores indicating more pain) [29] because of its importance in the metastatic setting, as well as cognitive function (EORTC QLQ‐C30 subscale, range 0–100, higher scores indicating a better function) [29] and psychological distress (PHQ‐4, range 0–12, higher scores indicating more distress) [30] which have been previously associated with QWL in patients with MBC [15]. Cancer treatment and characteristics were extracted from medical records, while sociodemographic factors were self‐reported.

### 2.5 Statistical Analysis

Statistical analyses were conducted using R version 4.4.0. We report two‐sided *p*‐values and considered a *p*‐value < 0.05 as statistically significant. All analyses were conducted on baseline data only, except for the investigation of exercise effects.

Firstly, we investigated factors associated with working hours per week. To account for the large number of zeros induced by those not working, we used a hurdle model (glmmTMB‐package) [31] where the random variable ‘working hours’ was modeled using two parts. First, the chances of working at all (0 vs. > 0 h) were modeled by a binary logistic component. If participants worked, the number of working hours was modeled with a truncated negative binomial component. The model included age, marital status (single/divorced/widowed vs. married), country, education [standardized across countries: basic (no formal schooling or only primary school completed) vs. middle (secondary school completed) vs. higher (high school completed) vs. academic education (college/university/post graduate degree)], cancer therapies received (yes/no: chemotherapy, endocrine therapy, targeted or immunotherapy, lymph node surgery), line of treatment (1st or 2nd line treatment vs. 3rd or later line), cognitive function, pain, psychological distress and fatigue. As only three participants had missing information on covariates, no imputation methods were applied, thus resulting in *N* = 284 participants included in the analysis. Residual plots were inspected using the DHARMa‐package.

For the subsample of recently working participants (*N* = 59), we initially fitted a general linear model for the overall score of QWL to identify associated factors. Since residual plots indicated some deviations from normality, we employed a median regression which uses conditional medians instead of means. The amount of physical, mental, assigned and management tasks at work was included in the model as well as cognitive function, psychological distress and fatigue. The only individual with a basic level of education was recoded as ‘middle education’ since including this single datapoint resulted in an invalid model fit.

To examine the effect of the exercise intervention on the volume of work over the 9‐month study period, we interpolated the working hours per month based on the values given at the four measurement time points and summed them. *N* = 13 participants were excluded from the analysis because of missing information on working hours, resulting in a final sample of *N* = 274. We calculated a hurdle model (using a logistic and truncated negative binomial distribution) with group (intervention vs. control group) as the independent variable, adjusting for baseline working hours as well as the stratification factors used for randomization, study center, and therapy line. To check for goodness‐of‐fit, residual plots were inspected with the help of the DHARMa package.

## 3 Results

### 3.1 Sample Characteristics

Of the 357 patients with MBC enrolled in the PREFERABLE‐EFFECT trial, the present analysis included those 287 patients who were within working age (age 18–65 years). The QLQW‐CS questionnaire was assessed as an add‐on to the EFFECT trial only in some study centres (i.e., Germany, the Netherlands, Spain, and Australia). This questionnaire was applicable only to patients who had a paid job and were actively working in the past 4 weeks, resulting in *N* = 59 at baseline. An overview of the participant flow can be found in Data S2.

Participants' baseline characteristics for the whole study sample and the subsample reporting on QWL can be found in Table 1.

**TABLE 1:** Sociodemographic, treatment‐related, and psychological characteristics of PREFERABLE‐EFFECT study participants at baseline.

	Working age participants^a^ *N* = 287 (100%)	QWL subsample^b^ *N* = 59 (100%)
Gender		
Female	285 (99.3%)	59 (100%)
Male	2 (0.7%)	0 (0.0%)
Age	51.4 (8.2)	51.1 (8.3)
Country of study participation		
Australia	26 (9.1%)	15 (25.4%)
Germany	79 (27.5%)	9 (15.3%)
The Netherlands	75 (26.1%)	33 (55.9%)
Poland	35 (12.2%)	0 (0.0%)
Spain	38 (13.2%)	2 (3.4%)
Sweden	34 (11.9%)	0 (0.0%)
Education		
Academic	159 (55.4%)	33 (55.9%)
Higher	65 (22.7%)	10 (16.9%)
Middle	57 (19.9%)	15 (25.4%)
Basic	6 (2.1%)	1 (1.7%)
Other	0 (0.0%)	0 (0.0%)
Marital status		
Married	205 (71.4%)	40 (67.8%)
Single/divorced/widowed	82 (28.6%)	19 (32.2%)
Completed treatments		
Primary breast surgery	189 (65.9%)	43 (72.9%)
Lymph node surgery	128 (44.6%)	31 (52.5%)
Chemotherapy	188 (65.5%)	40 (67.8%)
Radiotherapy	166 (57.8%)	36 (61.0%)
Endocrine therapy	158 (55.1%)	49 (66.1%)
Targeted/immune therapy	74 (25.8%)	12 (20.3%)
Lines of treatment		
1st or 2nd	216 (75.3%)	53 (89.8%)
3rd or later	71 (24.7%)	6 (10.2%)
Years since first breast cancer diagnosis	6.6 (5.4)	6.4 (5.3)
Depression^c^		
Yes	46 (16.0%)	7 (11.9%)
No	241 (84.0%)	52 (88.1%)
Anxiety^c^		
Yes	63 (22.0%)	12 (20.3%)
No	224 (78.1%)	47 (79.7%)
Quality of life		
Summary score^d^	73.5 (14.2)	79.2 (12.8)
Functional subscales^d^		
Physical	79.0 (15.9)	86.2 (10.6)
Role	70.3 (25.8)	76.8 (23.6)
Emotional	63.0 (23.9)	68.5 (20.8)
Cognitive	71.5 (25.2)	75.7 (24.6)
Social	65.3 (27.8)	71.8 (28.1)
Symptom scales^c^		
Fatigue	45.8 (24.3)	36.5 (22.3)
Nausea and vomiting	8.0 (14.2)	7.9 (15.9)
Pain	32.1 (24.5)	20.9 (18.9)
Dyspnea	25.8 (26.9)	15.8 (21.8)
Insomnia	38.2 (29.6)	37.9 (27.3)
Appetite loss	14.6 (24.2)	10.2 (21.7)
Constipation	14.5 (25.9)	11.3 (21.9)
Diarrhea	14.1 (26.3)	9.6 (18.6)
Financial difficulties	20.6 (28.8)	10.2 (25.8)
Fatigue (EORTC QLQ‐FA12)^c^		
Total	31.8 (19.7)	27.0 (19.1)
Cognitive	16.3 (20.9)	13.8 (17.3)
Physical	41.7 (24.1)	35.3 (23.4)
Emotional	29.2 (25.4)	23.35 (21.6)

*Note:* Depression and anxiety scales were categorized as normal or pathological according to Kroenke et al. [30].

^a^ Working age was defined as 18–65 years.

^b^ QWL was assessed at four study centres in Germany, Australia, the Netherlands, and Spain among the participants who reported having worked in the last 4 weeks.

^c^ Higher scores indicate a higher symptom burden.

^d^ Higher scores indicate a better functioning.

### 3.2 Employment Status

Information on employment status and work‐related variables of participants at baseline is displayed in Table 2.

**TABLE 2:** Work characteristics of the study participants at baseline.

	Working age participants *N* = 287 (100%)	QWL subsample^d^ *N* = 59 (100%)
Employed^a^	157 (54.7%)	59 (100%)
Actively working^b^	81 (51.6%)	43 (72.9%)
On sick leave	75 (45.3%)	16 (27.1%)
Unclear	1 (0.6%)	0
Not employed	130 (46.7%)	0
Permanently disabled	67 (51.4%)	0
Housewife/Houseman	23 (17.7%)	0
(Prematurely) retired	19 (14.6%)	0
Student	3 (2.3%)	0
Unemployed/Seeking work	4 (3.1%)	0
Unclear	14 (10.8%)	0
Working hours per week (if any)	27.3 (11.7)	23.7 (11.4)
Type of work^c^		
Physical work	2.3 (1.2)	2.4 (1.2)
Mental work	4.2 (0.9)	3.8 (0.9)
Management tasks	3.6 (1.1)	3.7 (1.0)
Carrying out assigned tasks	3.8 (1.0)	3.8 (1.0)

^a^ Employment was defined as having a paying job.

^b^ Participants reporting > 0 working hours were categorized as actively working.

^c^ Participants rated how much they performed each type of task at their work on a scale from *1 (never)* to *5 (always)*.

^d^ QWL was assessed at four study centres in Germany, Australia, the Netherlands, and Spain among the participants who reported having worked in the past 4 weeks.

The results of the hurdle model on working volume of participants can be found in Table 3. The binary part of the hurdle model on working hours per week indicated a significant association of pain with working (*p* = 0.014), with the odds of working decreasing with an increase in pain. Additionally, living in Spain (compared to Sweden) was associated with lower odds of working (*p* = 0.002). Being married compared to being single/divorced/widowed decreased the chance of working (*p* = 0.048), as did a higher line of treatment (compared to 1st or 2nd line of treatment), although not statistically significant (*p* = 0.059). Considering the number of working hours (truncated negative binomial model), having an academic education (*p* = 0.031) as well as a basic education (*p* = 0.040) compared to a middle level education was associated with an increase in working hours. Having received targeted/immune therapy was associated with fewer working hours compared to not having received these therapies (*p* = 0.013). Unexpectedly, psychological distress (as indicated by a higher PHQ‐4 total score) was positively associated with working hours (*p* < 0.001), but also better cognitive function tended to be associated with a higher number of working hours (*p* = 0.051).

**TABLE 3:** Results of the hurdle model on working hours at baseline.

	Binary logistic model: working at all (> 0 h vs. 0 h)			Truncated negative binomial model: number of working hours (if working at all)		
	Odds ratios (ORs)	95% CI	*p*	Incidence rate ratios (IRRs)	95% CI	*p*
Cognitive function^a^	1.01	0.86–1.19	0.898	1.06	1.00–1.13	0.051
Pain^a^	0.83	0.71–0.96	**0.014**	1.01	0.96–1.07	0.677
Fatigue^a^	0.85	0.68–1.09	0.194	1.00	0.92–1.07	0.909
Psychological distress^b^	0.94	0.81–1.09	0.405	1.08	1.04–1.13	**< 0.001**
Age	0.99	0.95–1.02	0.432	0.99	0.98–1.01	0.330
Marital status						
Married	0.52	0.27–1.00	**0.048**	1.00	0.82–1.22	0.993
Single/Divorced/Widowed	REF	REF	REF	REF	REF	REF
Education						
Basic	2.78	0.27–25.00	0.363	1.75	1.02–2.99	**0**.**040**
Middle	REF	REF	REF	REF	REF	REF
Higher	0.92	0.35–2.83	0.857	1.34	0.98–1.83	0.064
Academic	1.20	0.51–2.86	0.6733	1.37	1.03–1.82	**0.** **031**
Country						
Sweden	REF	REF	REF	REF	REF	REF
Australia	1.35	0.41–4.55	0.625	0.75	0.54–1.03	0.076
Germany	0.71	0.23–2.17	0.557	0.77	0.55–1.09	0.139
The Netherlands	0.43	0.14–1.27	0.125	0.77	0.55–1.06	0.110
Poland	0.93	0.29–3.03	0.905	0.94	0.69–1.26	0.667
Spain	0.08	0.02–0.39	**0.** **002**	0.58	0.33–1.01	0.054
Chemotherapy	0.90	0.46–1.79	0.766	1.07	0.87–1.32	0.528
Endocrine therapy	1.67	0.88–3.23	0.120	1.05	0.86–1.29	0.611
Targeted/immune therapy	0.88	0.41–1.88	0.757	0.75	0.60–0.94	**0.** **013**
Lymph node surgery	1.03	0.52–2.04	0.937	0.83	0.68–1.01	0.059
Line of treatment						
1st or 2nd	REF	REF	REF	REF	REF	REF
3rd or later	0.44	0.19–1.03	0.059	1.14	0.90–1.45	0.278

^a^ ORs and IRRs for cognitive function, pain, and fatigue are reported per 10‐point increase.

^b^ The degree of psychological distress is represented by the PHQ‐4 total score. REF indicates the reference category used in the analysis.

Participants' self‐reported work reductions and the provided reasons as well as wishes for the work arrangement are reported in Table 4. The most important reasons for having reduced work were fatigue, memory issues, and an inability to complete the required tasks at work, while participants expressed a need for less pressure to be as productive as before the disease and more flexible working hours.

**TABLE 4:** Work reductions and patient‐reported reasons and wishes of working‐age participants employed at baseline, sorted by frequency.

	Employed participants *N* = 157 (100%)
Having reduced working hours or work load in the past 3 months	48 (30.6%)
Self‐reported reasons why work was reduced (up to 3 answers were possible)	48 (100%)
Severe exhaustion/Fatigue	20 (41.7%)
Problems with concentration/memory	16 (33.3%)
I could no longer complete all my tasks	16 (33.3%)
Other things in life are more important to me now	14 (29.2%)
Pain	13 (27.0%)
Limited physical functioning	10 (20.8%)
I had the feeling that I could no longer cope with the pressure	9 (18.8%)
I am no longer able to coordinate my private and work‐related commitments^a^	8 (16.7%)
Other medical issues^b^	6 (12.5%)
My employer/colleagues did not show any understanding for my situation	3 (6.3%)
It is financially not necessary for me to work any longer	2 (4.2%)
Other	2 (4.2%)
Self‐reported wishes for job arrangement	48 (100%)
Less pressure to be as productive as before the disease	18 (37.5%)
More flexible working hours	14 (29.2%)
More time to complete tasks	6 (12.5%)
More support from colleagues	4 (8.3%)
More support with employment law issues	4 (8.3%)
Being able to talk openly about problems with managers or colleagues	4 (8.3%)
Being regarded as a fully valued member at workplace	3 (6.3%)
Change of tasks/job^b^	2 (4.2%)
Less responsibility^b^	1 (2.1%)
Other	4 (8.3%)

^a^ Two open answers were counted in this category.

^b^ This category was built from open answers.

### 3.3 Quality of Working Life

**FIGURE 1:**
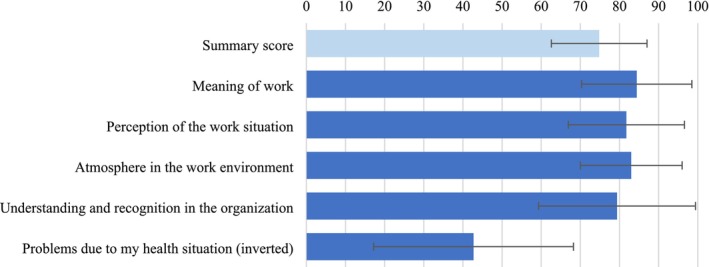
Standardized mean scores of the QWL subscales (0 = worst QWL to 100 = best QWL) and summary score as well as corresponding standard deviations at baseline.

With regard to QWL, the mean standardized score at baseline on the summary scale and subscales is displayed in Figure 1.

Regression on the QWL summary score indicated that a higher level of fatigue (*β* = −0.25, 95% CI [−0.40, −0.11], *p* = 0.001), more mentally strenuous work (*β* = −4.89, 95% CI [−7.04, −2.74], *p* < 0.001) and an academic education compared to a middle level of education (*β* = −9.23, 95% CI [−13.57, −4.89], *p* < 0.001) were associated with lower baseline QWL. On the other hand, a higher amount of management tasks was related to better QWL (*β* = 2.89, 95% CI [0.65, 5.13], *p* = 0.015). All other factors were not associated significantly with baseline QWL scores.

### 3.4 Exercise Intervention Effect

**FIGURE 2:**
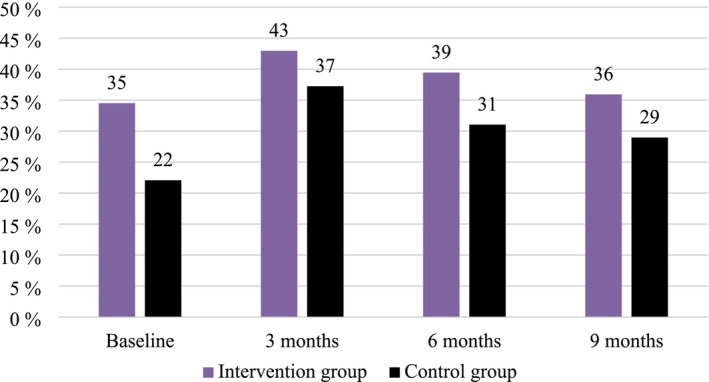
Percentage of participants actively working at the different assessment time points, by treatment group. Percentages are calculated based on the number of participants randomized to each group at baseline.

Figure 2 illustrates the numbers of participants actively working over the course of the study. It indicates that disparities between the two groups already existed at the start of the study. The number of working participants increased at 3 months and continuously decreased thereafter, regardless of the exercise intervention.

A hurdle model on the volume of work per month indicated no statistically significant association between the exercise intervention and working (yes vs. no) during the course of the study (OR = 0.71, 95% CI [0.36, 1.39], *p* = 0.312); nor was it associated with a higher number of working hours per month and participants who worked (IRR = 1.06, 95% CI [0.88, 1.29], *p* = 0.526).

## 4 Discussion

Knowledge about factors that impact the working life of patients with MBC is essential for improving their work capacity and QWL. Our results indicate that fatigue was the main reason to reduce working hours. Additionally, higher levels of pain emerged as the main determinant for not working. Furthermore, fatigue, doing more mental tasks, and an academic education were associated with lower QWL. Conversely, a higher number of working hours was linked to higher psychological distress. We did not observe any beneficial effects of the exercise intervention on the volume of work.

The employment rate of 55% of working age participants in the study was well within the range reported in other studies including patients with MBC, 21%–74% [6, 15]. The main factor associated with work status was pain, which was also reported as a reason for having reduced working hours. This confirms initial findings from previous studies [6, 32] and highlights the importance of reducing pain in patients with metastatic disease. Besides pain, fatigue was associated with reduced QWL and was the most commonly self‐reported reason for reducing working hours. These results align with previous findings on the detrimental effects of fatigue on work outcomes [3, 9]. Symptom management for fatigue is thus also important for preserving a patient's ability or willingness to work.

The impact of higher education and cognitive function on working life was ambiguous: On the one hand, both were related to more working hours, but a high proportion of cognitively challenging tasks and an academic education were associated with lower QWL. This might indicate that patients who had more years of education (e.g., white collar workers) are more likely to continue working, but at the same time they might be overstrained by their jobs. This idea is further supported by the finding that participants wished for less pressure to be as productive as before their disease, and the fact that several patients had reduced working hours because of an inability to comply with the demands being placed on them. In line, Chapman et al. [15] found better cognitive function and fewer depressive symptoms to be associated with higher QWL. We also found an association between higher levels of psychological distress with more working hours. Our data do not allow any causal statements. However, more working hours per week may have contributed to higher psychosocial distress for some patients. Management tasks posed an exception to cognitively challenging jobs in this study, with a higher proportion of management tasks going hand in hand with higher QWL, as observed by a previous study [17]. These jobs might come with a higher level of autonomy and might, therefore, be more compatible with workers' needs (e.g., by offering more flexibility). Past studies found that patients with cancer generally stay in the workforce longer if their employer is accommodating [33, 34, 35]. Our results point in a similar direction, indicating that less performance pressure or more flexible working time could help patients to maintain their previous work volume.

In contrast to previous studies conducted about a decade ago [3, 36], we did not find that chemotherapy was negatively associated with work. A reason might be that, today, chemotherapy is used somewhat more cautiously, and several side effects are now better managed than in the past. However, we found that targeted or immunotherapy was negatively associated with work, independent of treatment line. We are not aware of any other study reporting on this topic. The field of new targeted or immunotherapies is evolving rapidly, and some of their side effects (e.g., cardiotoxicity in the case of trastuzumab [37]) and negative associations with quality of life [38, 39] have already been documented. Future studies are needed to determine whether these findings can be replicated and explore specific underlying causes for reducing work in patients receiving these therapies.

In line with previous studies, single (or divorced/widowed) patients were more likely to be working compared to married patients [3, 9]. A potential reason may be a financial need, which may also apply to patients with low education working more hours per week. Sesto et al. [40] previously identified financial reasons as an important factor for patients with MBC to keep working. The fact that our Swedish participants were more likely to work than participants in Spain is surprising since a previous Swedish study reported few working days in employed patients with MBC [41]. One potential reason for country differences might be differences in national health and labor policies. Future research will need to further examine these country differences before any conclusions can be drawn.

Our trial did not indicate that the exercise intervention promoted working or a higher volume of work in patients with MBC. At first glance, this is surprising since the trial showed a significant effect of the exercise intervention on pain and fatigue [42] that, in turn, were associated with lower QWL and a reduction of work. On the other hand, there are many other factors that might influence how much people work, e.g., financial factors or local policies. These factors are probably less amenable to being influenced by exercise. Nevertheless, it is worth noting that participating in the exercise intervention did not have a negative impact on participants' work. Since the exercise sessions took place twice weekly, during the day, this could have interfered with participants' working hours. However, this was not observed. There was an increasing rate of working participants in both groups during the first three months of the trial. This may have been due, in part, to recruitment during active therapy and therefore temporary sick leave. Yet, it may also indicate that study participants wanted to take control of their lives and try to return to normal life, in addition to increasing their physical activity.

## 5 Strengths and Limitations

In general, this analysis was limited by its exploratory nature, as work‐related questions were only secondary endpoints of the PREFERABLE‐EFFECT trial. Likewise, other potentially important factors like income or work ability were not assessed within the study. Participants were thus recruited regardless of whether they actually wanted to continue working or increase their working hours. At the same time, the sample consists only of patients who were willing to participate in an exercise trial. This might limit generalizability since participants might have been fitter, better educated, or had more financial security than the average patient. Furthermore, most of our analyses did not allow for causal inferences since they were based on cross‐sectional data. Additionally, the sample for the QWL analysis was small. The heterogeneity of our sample (e.g., participants came from six countries) might have reduced the statistical power to find significant associations. At the same time, the multinational nature of the sample is a strength, as this improves the generalizability of our results. We are aware of only one previous study examining QWL in patients with MBC. Therefore, our study provides new knowledge for improving the work situation of patients with MBC to support them to continue working as long and at the highest QWL as possible.

## 6 Conclusions

Our study results suggest that job expectations and demands may need to be adapted to the needs and abilities of patients diagnosed with MBC. Employers should consider supporting their employees by offering more flexible working hours and making tasks more achievable. Patients who continue working may need support in managing cognitive issues. Additionally, healthcare providers should be aware that symptom management, especially for pain and fatigue, may be important for maintaining patients' ability to work.

## Author Contributions

**Alina Kias:** conceptualization, data curation, formal analysis, visualization, writing – original draft, writing – review and editing. **Martina E. Schmidt:** conceptualization, funding acquisition, formal analysis, writing – original draft, writing – review and editing. **Anouk E. Hiensch:** data curation, investigation, project administration, writing – review and editing. **Dorothea Clauss:** investigation, writing – review and editing. **Evelyn M. Monninkhof:** investigation, writing – review and editing. **Mireia Pelaez:** investigation, writing – review and editing. **Jon Belloso:** conceptualization, funding acquisition, investigation, writing – review and editing. **Nadira Gunasekara:** investigation, writing – review and editing. **Maike G. Sweegers:** investigation, writing – review and editing. **Mark Trevaskis:** investigation, writing – review and editing. **Helene Rundqvist:** conceptualization, funding acquisition, investigation, writing – review and editing. **Jana Müller:** writing – review and editing, investigation. **Joachim Wiskemann:** writing – review and editing. **Elsken van der Wall:** conceptualization, funding acquisition, writing – review and editing. **Neil K. Aaronson:** conceptualization, funding acquisition, writing – review and editing. **Milena Lachowicz:** conceptualization, writing – review and editing. **Ander Urruticoechea:** conceptualization, funding acquisition, writing – review and editing. **Eva M. Zopf:** conceptualization, funding acquisition, writing – review and editing. **Wilhelm Bloch:** conceptualization, funding acquisition, writing – review and editing. **Martijn M. Stuiver:** conceptualization, funding acquisition, writing – review and editing. **Yvonne Wengström:** conceptualization, funding acquisition, writing – review and editing. **Anne M. May:** conceptualization, funding acquisition, project administration, supervision, writing – review and editing. **Karen Steindorf:** conceptualization, funding acquisition, project administration, supervision, writing – original draft, writing – review and editing.

## Ethics Statement

The study was conducted in accordance with the standards of Good Clinical Practice and the Declaration of Helsinki. The conduct of the study was approved by the local ethical review boards of all participating institutions.

## Consent

Informed consent was obtained from all individual participants included in the study.

## Conflicts of Interest

The authors declare no conflicts of interest.

## Supporting information


**Data S1.** Supplement A. Questionnaire used to ask participants about their current work situation.

**Data S2.** Supplement B. Overview of (sub)samples analyzed.

## Data Availability

The data that support the findings of this study are available from the corresponding author upon reasonable request.

## References

[1] A. Welt , S. Bogner , M. Arendt , et al., “Improved Survival in Metastatic Breast Cancer: Results From a 20‐Year Study Involving 1033 Women Treated at a Single Comprehensive Cancer Center,” Journal of Cancer Research and Clinical Oncology 146, no. 6 (2020): 1559–1566, 10.1007/s00432-020-03184-z. PMC7230039 32189107

[2] M. Taskindoust , S. M. Thomas , S. L. Sammons , et al., “Survival Outcomes Among Patients With Metastatic Breast Cancer: Review of 47,000 Patients,” Annals of Surgical Oncology 28, no. 12 (2021): 7441–7449, 10.1245/s10434-021-10227-3. 34050430 PMC8530869

[3] T. Islam , M. Dahlui , H. A. Majid , et al., “Factors Associated With Return to Work of Breast Cancer Survivors: A Systematic Review,” BMC Public Health 14, no. 3 (2014): S8, 10.1186/1471-2458-14-S3-S8. PMC4251139 25437351

[4] M. E. Schmidt , S. Scherer , J. Wiskemann , and K. Steindorf , “Return to Work After Breast Cancer: The Role of Treatment‐Related Side Effects and Potential Impact on Quality of Life,” European Journal of Cancer Care 28, no. 4 (2019): e13051, 10.1111/ecc.13051. 31033073

[5] D. Hernandez and M. Schlander , “Income Loss After a Cancer Diagnosis in Germany: An Analysis Based on the Socio‐Economic Panel Survey,” Cancer Medicine 10, no. 11 (2021): 3726–3740, 10.1002/cam4.3913. PMC8178494 33973391

[6] K. D. Lyons , R. M. Newman , M. Sullivan , M. Pergolotti , B. Braveman , and A. L. Cheville , “Employment Concerns and Associated Impairments of Women Living With Advanced Breast Cancer,” Archives of Rehabilitation Research and Clinical Translation 1, no. 1 (2019): 100004, 10.1016/j.arrct.2019.100004. 33543044 PMC7853337

[7] B. Porro , A. Michel , C. Zinzindohoué , et al., “Quality of Life, Fatigue and Changes Therein as Predictors of Return to Work During Breast Cancer Treatment,” Scandinavian Journal of Caring Sciences 33, no. 2 (2019): 467–477, 10.1111/scs.12646. 30664270

[8] J. A. Hansen , M. Feuerstein , L. C. Calvio , and C. H. Olsen , “Breast Cancer Survivors at Work,” Journal of Occupational and Environmental Medicine 50, no. 7 (2008): 777–784, 10.1097/JOM.0b013e318165159e. 18617833

[9] R. A. Cocchiara , I. Sciarra , V. D'Egidio , et al., “Returning to Work After Breast Cancer: A Systematic Review of Reviews,” Work 61, no. 3 (2018): 463–476, 10.3233/WOR-182810. 30400123

[10] M. K. Lee , H. S. Kang , K. S. Lee , and E. S. Lee , “Three‐Year Prospective Cohort Study of Factors Associated With Return to Work After Breast Cancer Diagnosis,” Journal of Occupational Rehabilitation 27, no. 4 (2017): 547–558, 10.1007/s10926-016-9685-7. 27858198

[11] R. R. Bouknight , C. J. Bradley , and Z. Luo , “Correlates of Return to Work for Breast Cancer Survivors,” Journal of Clinical Oncology 24, no. 3 (2006): 345–353, 10.1200/jco.2004.00.4929. 16421415

[12] V. S. Blinder , S. Patil , A. Thind , et al., “Return to Work in Low‐Income Latina and Non‐Latina White Breast Cancer Survivors: A 3‐Year Longitudinal Study,” Cancer 118, no. 6 (2011): 1664–1674, 10.1002/cncr.26478. 22009703 PMC3263326

[13] H. Tavan , A. Azadi , and Y. Veisani , “Return to Work in Cancer Patients: A Systematic Review and Meta‐Analysis,” Indian Journal of Palliative Care 25, no. 1 (2019): 147–152, 10.4103/IJPC.IJPC_114_18. 30820118 PMC6388592

[14] M. de Jong , S. J. Tamminga , R. J. J. van Es , M. H. W. Frings‐Dresen , and A. de Boer , “The Quality of Working Life Questionnaire for Cancer Survivors (QWLQ‐CS): Factorial Structure, Internal Consistency, Construct Validity and Reproducibility,” BMC Cancer 18, no. 1 (2018): 66, 10.1186/s12885-017-3966-1. PMC5763640 29321006

[15] B. Chapman , E. A. Grunfeld , and N. Derakshan , “Quality of Working Life Can Protect Against Cognitive and Emotional Vulnerability in Women Living With Metastatic Breast Cancer: A Cross‐Sectional Study,” Journal of Cancer Survivorship 17, no. 5 (2023): 1295–1308, 10.1007/s11764-022-01169-0. 35038120 PMC8761843

[16] J. Jin , “Factors Associated With the Quality of Work Life Among Working Breast Cancer Survivors,” Asia‐Pacific Journal of Oncology Nursing 9, no. 2 (2022): 97–104, 10.1016/j.apjon.2021.11.005. 35529416 PMC9072182

[17] M. de Jong , S. J. Tamminga , M. H. Frings‐Dresen , and A. G. de Boer , “Quality of Working Life of Cancer Survivors: Associations With Health‐ and Work‐Related Variables,” Supportive Care in Cancer 25, no. 5 (2017): 1475–1484, 10.1007/s00520-016-3549-8. PMC5378750 28019005

[18] J. H. Jin and E. J. Lee , “Structural Equation Model of the Quality of Working Life Among Cancer Survivors Returning to Work,” Asian Nursing Research 15, no. 1 (2021): 37–46, 10.1016/j.anr.2020.10.002. 33253927

[19] C. S. Cleeland , M. Mayer , N. A. Dreyer , et al., “Impact of Symptom Burden on Work‐Related Abilities in Patients With Locally Recurrent or Metastatic Breast Cancer: Results From a Substudy of the VIRGO Observational Cohort Study,” Breast 23, no. 6 (2014): 763–769, 10.1016/j.breast.2014.08.004. 25193423

[20] N. Algeo , K. Bennett , and D. Connolly , “Rehabilitation Interventions to Support Return to Work for Women With Breast Cancer: A Systematic Review and Meta‐Analysis,” BMC Cancer 21, no. 1 (2021): 895, 10.1186/s12885-021-08613-x. 34353286 PMC8340442

[21] J. Hoving , M. Broekhuizen , and M. Frings‐Dresen , “Return to Work of Breast Cancer Survivors: A Systematic Review of Intervention Studies,” BMC Cancer 9, no. 1 (2009): 117, 10.1186/1471-2407-9-117. 19383123 PMC2678275

[22] A. G. de Boer , T. K. Taskila , S. J. Tamminga , M. Feuerstein , M. H. Frings‐Dresen , and J. H. Verbeek , “Interventions to Enhance Return‐To‐Work for Cancer Patients,” Cochrane Database of Systematic Reviews 2015, no. 9 (2015): CD007569, 10.1002/14651858.CD007569.pub3. 26405010 PMC6483290

[23] T. N. Wilson , A. Nambiema , B. Porro , et al., “Effectiveness of Physical Activity Interventions on Return to Work After a Cancer Diagnosis: A Systematic Review and Meta‐Analysis,” Journal of Occupational Rehabilitation 33, no. 1 (2023): 4–19, 10.1007/s10926-022-10052-9. 35779184 PMC10025244

[24] S. Zhou , G. Chen , X. Xu , et al., “Comparative Efficacy of Various Exercise Types on Cancer‐Related Fatigue for Cancer Survivors: A Systematic Review and Network Meta‐Analysis of Randomized Controlled Trials,” Cancer Medicine 14, no. 7 (2025): e70816, 10.1002/cam4.70816. 40145635 PMC11948276

[25] A. E. Hiensch , A. Schouten , E. M. Monninkhof , et al., “Cost‐Utility Analysis of a Supervised Exercise Program for Patients With Metastatic Breast Cancer in the PREFERABLE‐EFFECT Randomized Controlled Trial (RCT),” Journal of Clinical Oncology 42, no. 16 (2024): 11121. 10.1200/JCO-24-01441 PMC11974635 39805062

[26] A. E. Hiensch , E. M. Monninkhof , M. E. Schmidt , et al., “Design of a Multinational Randomized Controlled Trial to Assess the Effects of Structured and Individualized Exercise in Patients With Metastatic Breast Cancer on Fatigue and Quality of Life: The EFFECT Study,” Trials 23 (2022): 610, 10.1186/s13063-022-06556-7. PMC9335464 35906659

[27] S. Kecke , J. Ernst , J. Einenkel , S. Singer , and A. Hinz , “Psychometric Properties of the Fatigue Questionnaire EORTC QLQ‐FA12 in a Sample of Female Cancer Patients,” Journal of Pain and Symptom Management 54, no. 6 (2017): 922–928, 10.1016/j.jpainsymman.2017.08.007. 28807705

[28] R. Hamood , H. Hamood , I. Merhasin , and L. Keinan‐Boker , “Work Transitions in Breast Cancer Survivors and Effects on Quality of Life,” Journal of Occupational Rehabilitation 29, no. 2 (2019): 336–349, 10.1007/s10926-018-9789-3. 29948472

[29] N. K. Aaronson , S. Ahmedzai , B. Bergman , et al., “The European Organization for Research and Treatment of Cancer QLQ‐C30: A Quality‐Of‐Life Instrument for Use in International Clinical Trials in Oncology,” Journal of the National Cancer Institute 85, no. 5 (1993): 365–376, 10.1093/jnci/85.5.365. 8433390

[30] K. Kroenke , R. L. Spitzer , J. B. W. Williams , and B. Löwe , “An Ultra‐Brief Screening Scale for Anxiety and Depression: The PHQ–4,” Psychosomatics 50, no. 6 (2009): 613–621, 10.1016/s0033-3182(09)70864-3. 19996233

[31] C. X. Feng , “A Comparison of Zero‐Inflated and Hurdle Models for Modeling Zero‐Inflated Count Data,” Journal of Statistical Distributions and Applications 8, no. 1 (2021): 8, 10.1186/s40488-021-00121-4. 34760432 PMC8570364

[32] E. Cox‐Martin , A. Anderson‐Mellies , V. Borges , and C. Bradley , “Chronic Pain, Health‐Related Quality of Life, and Employment in Working‐Age Cancer Survivors,” Journal of Cancer Survivorship 14, no. 2 (2020): 179–187, 10.1007/s11764-019-00843-0. 31828603 PMC7473420

[33] V. Blinder , C. Eberle , S. Patil , F. M. Gany , and C. J. Bradley , “Women With Breast Cancer Who Work for Accommodating Employers More Likely to Retain Jobs After Treatment,” Health Affairs (Millwood) 36, no. 1 (2017): 274–281, 10.1377/hlthaff.2016.1196. PMC5559299 28167716

[34] K. Bilodeau , M. M. Gouin , A. Fadhlaoui , and B. Porro , “Supporting the Return to Work of Breast Cancer Survivors: Perspectives From Canadian Employer Representatives,” Journal of Cancer Survivorship 18, no. 4 (2023): 1384–1392, 10.1007/s11764-023-01382-5. 37140676 PMC10157121

[35] M. D. Thomson and L. A. Siminoff , “Managing Work and Cancer Treatment: Experiences Among Survivors of Hematological Cancer,” Cancer 124, no. 13 (2018): 2824–2831, 10.1002/cncr.31375. 29660822 PMC6070342

[36] P. van Muijen , N. L. Weevers , I. A. Snels , et al., “Predictors of Return to Work and Employment in Cancer Survivors: A Systematic Review,” European Journal of Cancer Care 22, no. 2 (2013): 144–160, 10.1111/ecc.12033. 23279195

[37] A. Kichloo , M. Albosta , D. Dahiya , et al., “Systemic Adverse Effects and Toxicities Associated With Immunotherapy: A Review,” World Journal of Clinical Oncology 12, no. 3 (2013): 150–163, 10.5306/wjco.v12.i3.150. PMC7968107 33767971

[38] T. U. Schulz , S. Zierold , M. M. Sachse , et al., “Persistent Immune‐Related Adverse Events After Cessation of Checkpoint Inhibitor Therapy: Prevalence and Impact on Patients' Health‐Related Quality of Life,” European Journal of Cancer 176 (2022): 88–99, 10.1016/j.ejca.2022.08.029. 36198246

[39] D. C. Guven , M. S. Thong , and V. Arndt , “Survivorship Outcomes in Patients Treated With Immune Checkpoint Inhibitors: A Scoping Review,” Journal of Cancer Survivorship 19 (2024): 806–845, 10.1007/s11764-023-01507-w. 38175366 PMC12081552

[40] M. E. Sesto , C. B. Carroll , X. Zhang , et al., “Unmet Needs and Problems Related to Employment and Working as Reported by Survivors With Metastatic Breast Cancer,” Supportive Care in Cancer 30, no. 5 (2022): 4291–4301, 10.1007/s00520-021-06755-z. 35088147 PMC8959021

[41] A. Johnsson , N. A. Kiani , S. A. M. Gernaat , U. Wilking , I. Shabo , and E. Hedayati , “Planning for Return to Work During the First Year After Breast Cancer Metastasis: A Swedish Cohort Study,” Cancer Medicine 12, no. 9 (2023): 10840–10850, 10.1002/cam4.5752. 36880198 PMC10225211

[42] A. E. Hiensch , J. Depenbusch , M. E. Schmidt , et al., “Supervised, Structured and Individualized Exercise in Metastatic Breast Cancer: A Randomized Controlled Trial,” Nature Medicine 30 (2024): 2957–2966, 10.1038/s41591-024-03143-y. PMC11485212 39054374

