# Supplementary material for: Factors Associated With Employment and Quality of Working Life in Patients With Metastatic Breast Cancer
Source: Cancer Med. 2025 Jul 27;14(15):e71074. doi: 10.1002/cam4.71074 (PMC12296696; doi:10.1002/cam4.71074)
Supplement: Supplementary file 1 — Data S1. Supplement A. Questionnaire used to ask participants about their current work situation. [file CAM4-14-e71074-s001.docx]

## Work questionnaire

| 1. **What does your current occupation involve?** | **Never (1)** | **Rarely** | **Sometimes** | **Often** | **Always (5)** |
| --- | --- | --- | --- | --- | --- |
| Physically strenuous tasks |  |  |  |  |  |
| Mentally strenuous tasks |  |  |  |  |  |
| Management and leadership related tasks such as planning, organizing, decision making and monitoring |  |  |  |  |  |
| Perform assigned tasks |  |  |  |  |  |

1. **Have you terminated your employment in the past 3 months or reduced the number of working hours or the workload?**

- Yes 🡪 c
- No 🡪 End

1. **What were the main reasons for this (select up to 3 reasons)?**

- Pain
- Severe exhaustion/ Fatigue
- Problems with concentration/ memory
- Impaired physical capacity
- I can no longer perform all occupational tasks/ I can no longer work at full capacity
- I am no longer able to coordinate my private and work-related commitments
- I had the feeling that I could no longer cope with the pressure
- My employer/ colleagues did not show any understanding for my situation
- It is financially not necessary for me to work any longer
- Other things in life are more important to me now
- Other reasons: ___________________________

1. **What do you require / would you like to see at work / from your employer (select up to 3)?**

- More flexible working hours
- More support from my colleagues
- More time to complete my tasks
- Less pressure to be as productive as before the disease
- More support regarding working rights
- Being regarded as a fully valued member at my workplace
- Being able to talk openly about problems with my managers or colleagues
- Other:__________________________
