# Supplementary figures and images for: Factors Associated With Employment and Quality of Working Life in Patients With Metastatic Breast Cancer
Source: Cancer Med. 2025 Jul 27;14(15):e71074. doi: 10.1002/cam4.71074 (PMC12296696; doi:10.1002/cam4.71074)

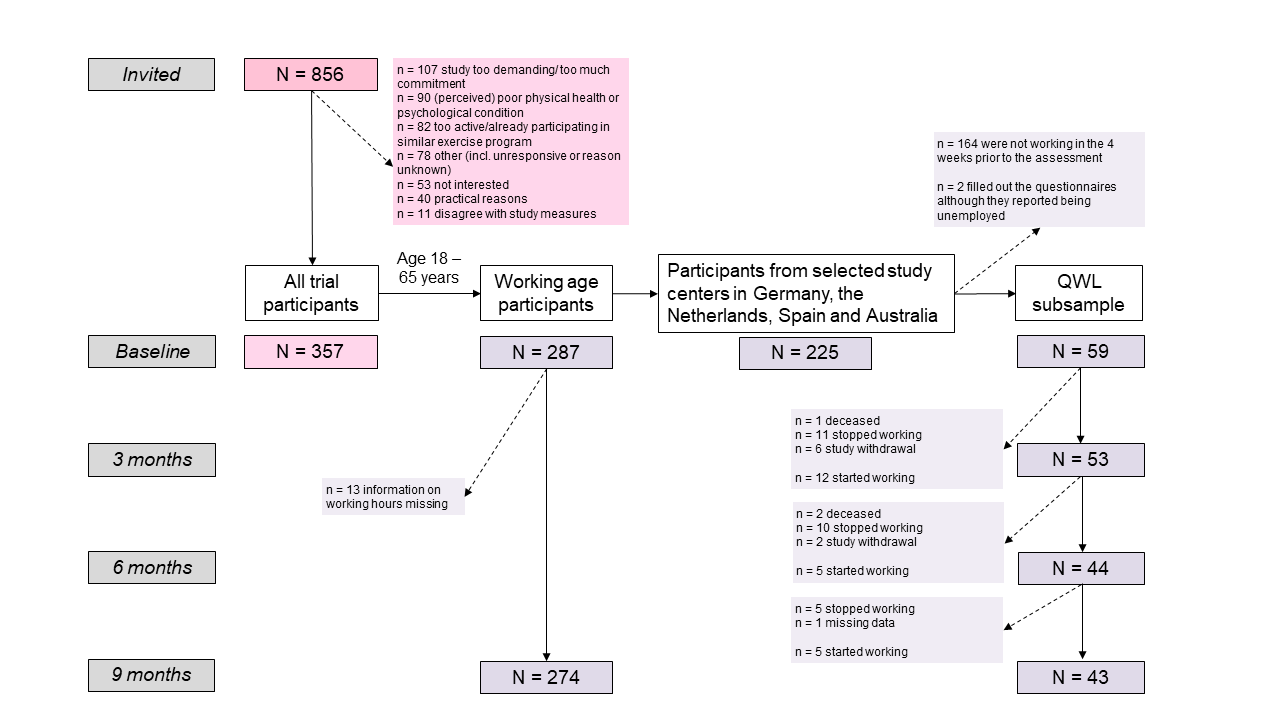

Supplement: Supplementary file 2 — Data S2. Supplement B. Overview of (sub)samples analyzed. [file CAM4-14-e71074-s002.png]
